# Tears of biceps femoris, semimembranosus, and semitendinosus are not equal—a new individual muscle-tendon concept in athletes

**DOI:** 10.1177/1457496920984274

**Published:** 2021-02-21

**Authors:** Lasse Lempainen, Jussi Kosola, Ricard Pruna, Juha-Jaakko Sinikumpu, Xavier Valle, Olli Heinonen, Sakari Orava, Nicola Maffulli

**Affiliations:** Sports Trauma Research Unit, Hospital Mehiläinen NEO, Joukahaisenkatu 6, Turku, FI 20520, Finland; Department of Physical Activity and Health, Paavo Nurmi Centre, University of Turku, Turku, Finland; Department of Physical Activity and Health, Paavo Nurmi Centre, University of Turku, Turku, Finland; Department of Surgery, Kanta-Häme Central Hospital, Hämeenlinna, Finland; FC Barcelona, Medical Services, FIFA Center of Excellence, Barcelona, Spain; Department of Children and Adolescents, PEDEGO unit and MRC Oulu, Oulu University Hospital and University of Oulu, Oulu, Finland; FC Barcelona, Medical Services, FIFA Center of Excellence, Barcelona, Spain; Department of Physical Activity and Health, Paavo Nurmi Centre, University of Turku, Turku, Finland; Sports Trauma Research Unit, Hospital Mehiläinen NEO, Turku, Finland; Department of Physical Activity and Health, Paavo Nurmi Centre, University of Turku, Turku, Finland; Department of Musculoskeletal Disorders, University of Salerno School of Medicine, Surgery and Dentistry, Salerno, Italy Centre for Sports and Exercise Medicine, Queen Mary University of London, London, UK; Institute of Science and Technology in Medicine, Keele University School of Medicine, Stoke on Trent, UK

**Keywords:** Hamstring injury, surgical treatment, athlete, tendon, biceps femoris, semitendinosus, semimembranosus

## Abstract

**Objectives:**

Hamstring injuries are common and can now be accurately diagnosed. In addition, novel surgical indications have been introduced. However, evidence-based guidelines on the hamstring injuries in management of top-level athletes are missing.

**Methods:**

The management methods and outcomes of treatment are classically based on relatively small case series. We discuss a novel concept based on the fact that each tendon of the hamstrings muscle should be managed in an individual fashion. Furthermore, suitable indications for hamstring surgery in athletes are introduced.

**Results:**

The present study introduces modern treatment principles for hamstring injury management. Typical clinical and imagining findings as well as surgical treatment are presented based on a critical review of the available literature and personal experience.

**Conclusions:**

Hamstring injuries should not be considered to be all equal given the complexity of this anatomical region: The three separate tendons are different, and this impacts greatly on the decision-making process and outcomes in athletes.

## Introduction

The three major muscles forming the hamstring muscle group are biceps femoris (BF), semimembranosus (SM), and semitendinosus (ST).^1^ These individual muscles—BF, SM, ST—serve different functions and exert a specific role and a specific contact times while walking, running, or turning, although their anatomy partially overlaps proximally.^2^

Typically, hamstring injuries are classified according to the location of the injury (i.e. proximal, middle, or distal), and the muscles are often considered as part of the hamstring complex.^3,4^ Injuries of the proximal hamstrings can range from partial to complete tears, and one, two, or all three tendons may avulse from the ischial tuberosity.^5-8^ The higher the grade of injury, the more likely it is for an athlete to undergo operative treatment.^5,6^

Hamstring injuries are common in running, sprinting, and jumping events and especially common in soccer^9^: up to five hamstring strains per club per season have been reported.^10^ Most of these injuries can be managed conservatively, and surgery is generally not necessary.^3,11,12^ However, the optimal treatment of these injuries is still largely unknown, and recurrent hamstring injuries occur far too often.^13^

The aim of this study is to highlight the importance of the individuality of every single hamstring muscle-tendon unit to allow to make a treatment decision in hamstring injuries in athletes. Hamstring tears are not all equal: this concept simplifies making the treatment decision, highlights the importance of each single muscle—tendon unit, and should optimize the treatment results in athletes. We demonstrate the relevance of this concept by showing different individual tendon injuries and giving perspective for their subsequent treatment.

### Different functional anatomy of each hamstrings

**Fig. 1.**
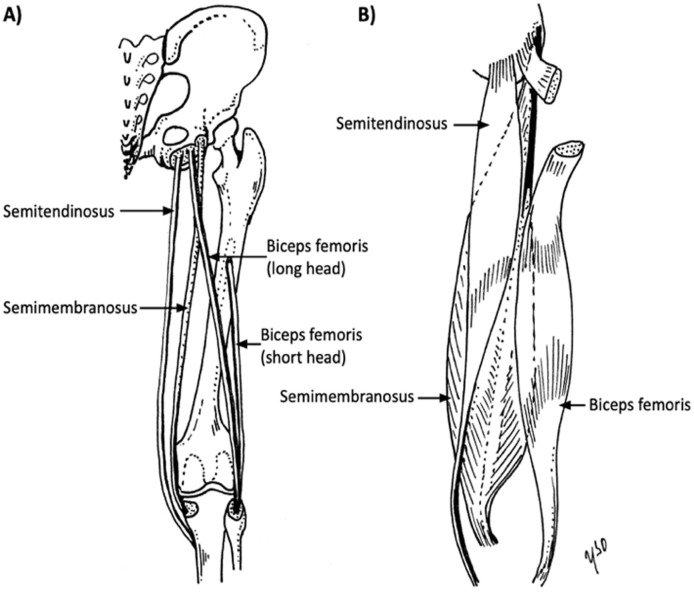
The illustrative drawing of each individual hamstring structure: A) tendons, B) muscles.

The hamstring muscle complex cross the hip and the knee (Fig. 1). With the exception of the short head of BF, the muscles of the posterior aspect of the thigh are mainly hip extensors and knee flexors with subtle rotational features. Distally, these muscles act as horse reins for rotational stabilization and reinforce the capsule while stabilizing the posterior structures such as menisci.^14^

The short head of BF originates at the linea aspera of the posterior aspect of the femur, and the ischial tuberosity is the origin of all the other hamstring muscles.^1^ The SM originates at the anterolateral portion of the ischial tuberosity, and the ST and the long head of the BF originate at the posteromedial parts with a partly conjoined tendon.^15^ Recently, more interest has focused on the central tendon of the hamstring muscles.^16,17^ The central tendons originate at their relevant muscle belly and extend along the entire length of the muscle.^1,18^

The ST has the shortest proximal tendon and the smallest physiological cross-sectional area (8.08 cm^2^) of the hamstring muscle group.^1^ It attaches distally at the superior aspect of the medial tibiae becoming part of the pes anserinus.

The SM is the largest muscle of the posterior thigh, with the longest proximal tendon. Its proximal insertion is connected to the ischial tuberosity with a broad aponeurosis. The distal portion consists of tendinous branches joined to the popliteal fascia and the oblique popliteal ligament and attached to the posterior portion of the medial tibial condyle.^14^

The BF exerts more force compared with ST or SM.^19^ Distally, the BF tendon inserts at the anterior and posterior border of the proximal portion of the fibular head.^20^

### Injury patterns

The typical mechanism of hamstring injuries is an eccentric muscle contraction accompanied by forced hyperflexion of the hip and extension of the knee.^21,22^ Patients often report a “popping” sensation when sustaining this injury.^23^ A common mechanism for the most severe hamstring injury is a rapid flexion of the hip during an ipsilateral eccentric knee extension, often from a fall while, for example, water skiing resulting in a front split, with a (complete) proximal hamstring rupture. A proximal isolated SM rupture can occur in extreme positions during the extended hip flexion movements performed by ballet dancers.^24^ Isolated SM strain can also occur in slow, apparently well-controlled, stretching exercises performed to the limit of the range of motion when isolated proximal BF injury occurs often, for example, while sprinting.

While these injury patterns are fairly typical in proximal hamstring tears, the distal parts of hamstrings may suffer an injury in a different way. The distal ST has been reported to tear during eccentric hamstring load during high-speed running, when the hamstrings are maximally activated.^25^ At this time, a switch from an eccentric to a concentric muscle contraction mode, with the individual muscles approaching their peak length, makes them most vulnerable to injury.

### Treatment decision when hamstring injury occurs—individual muscle concept

Clear evidence-based guidelines concerning proximal isolated injuries of ST, SM, and BF are not available in the current literature. Studies have introduced injury types which could correlate to poorer prognosis, and therefore treatment algorithms points toward to operative or nonoperative treatments have been made.^26,27^ However, the current literature does not consider these hamstring muscles or tendons individually.

**Fig. 2.**
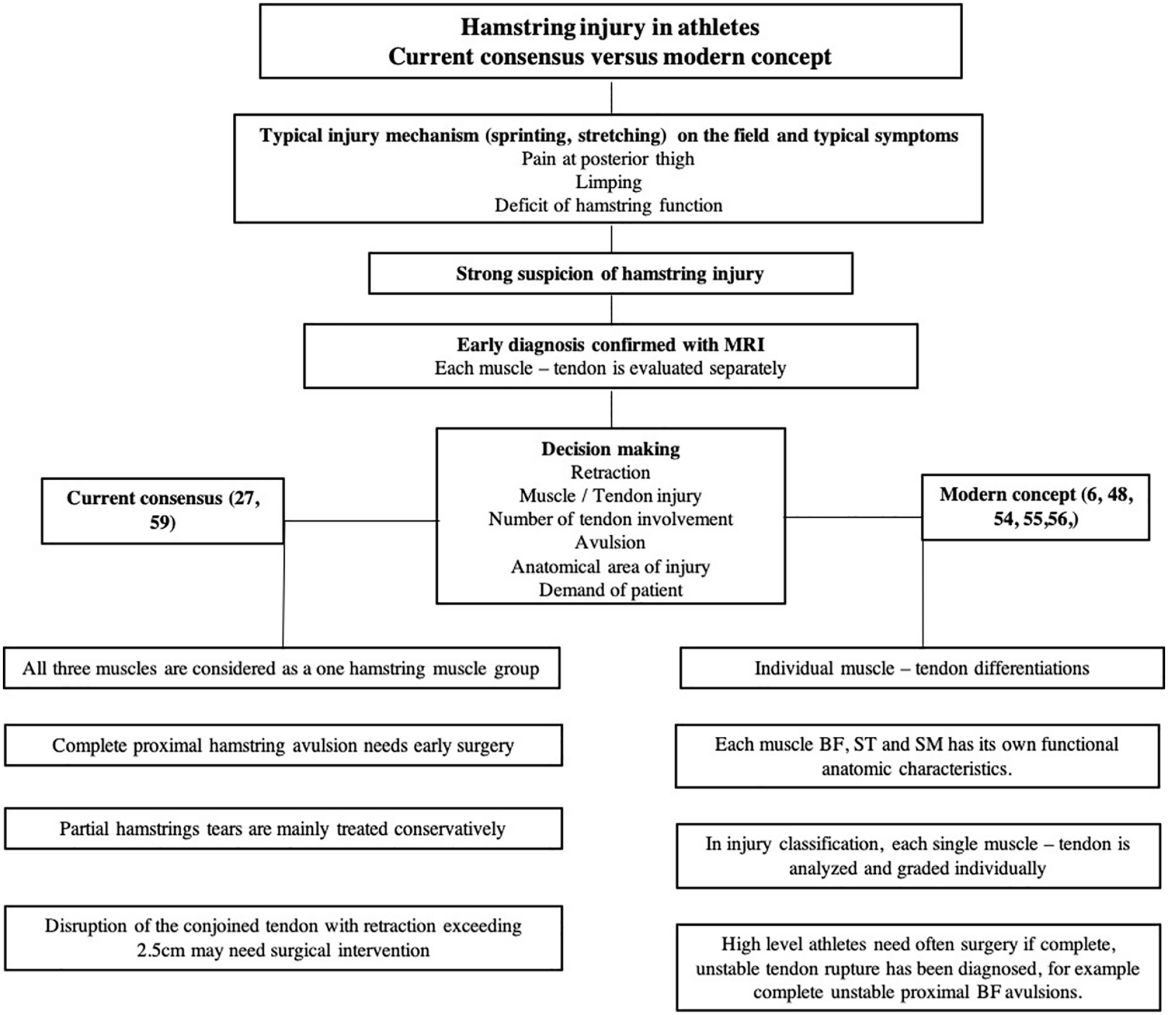
Comparison between current and modern treatment algorithms based on the present literature.

The first step in decision making is to formulate an exact diagnosis (Fig. 2). Clinical findings, mechanism of injury, and patient’s history lead to the suspicion of a hamstring injury^28,29^ which is typically verified by magnetic resonance imaging (MRI). MRI allows to detect the location and extent of the injury, the presence of fluid collection, and which muscle and tendon structures are involved.^30,31^ At times, a repeat MRI is needed 2 weeks after injury to obtain more detailed information.^17^ Injuries with a good prognosis after appropriate conservative treatment include injuries at myotendinous junction and low-grade muscle tears.^28^ A hamstring injury is a risk factor for chronic and recurrent hamstring injuries^28^; thus, treatment should be well planned, and patients closely followed.^32^ Other possible risk factors include age, untreated muscle strength imbalance, and reduced flexibility.^33-38^

#### Proximal tendon

**Fig. 3.**
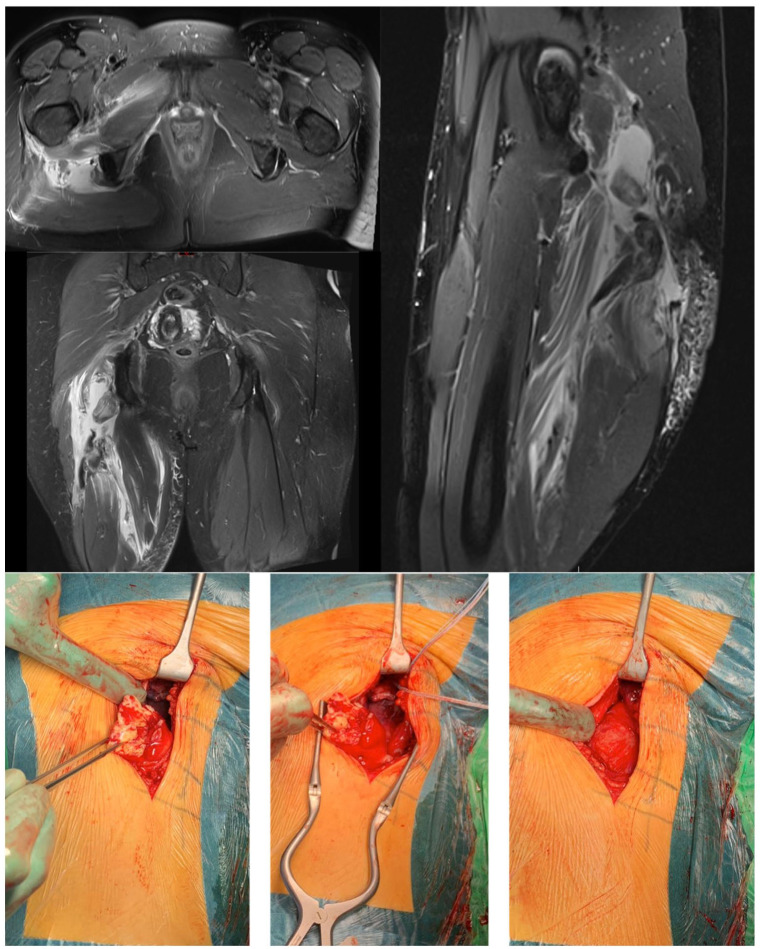
Complete three-tendon proximal hamstring rupture with a clear retraction at the right side.

**Fig. 4.**
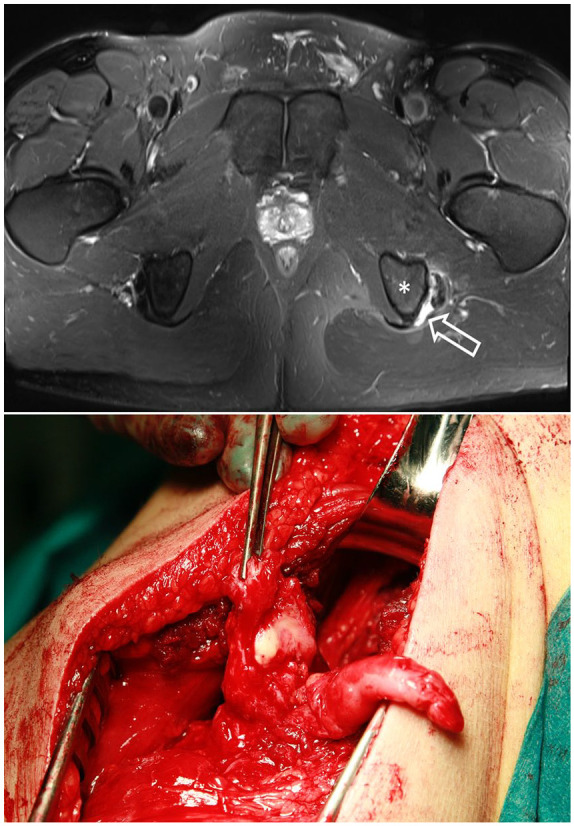
Perioperative image of the complete proximal two-tendon (BF and ST)—rupture. Arrow showing the ruptured area.

Recently, surgical treatment has become more popular for the management of hamstring injuries.^27^ If a tendon avulses from the ischial tuberosity, the degree of retraction seems to be associated with a poorer outcome^39^: a retraction greater than 2 cm is a classical indication for operative treatment in athletes. Retrospective data show that, in 25 patients, conservative and surgical management eventually resulted in a similar acceptable outcome, but initial nonoperative treatment led to conversion to surgery in 40% of the patients.^40^ Current literature regarding the management of one- or two-tendon avulsions is largely lacking, but most of these patients with symptomatic incomplete hamstring avulsions who are unresponsive to conservative management improved after surgical reinsertion.^41^ Early surgical treatment is recommended in complete proximal three-tendon hamstring avulsions (Fig. 3) and two-tendon (BF + ST / BF + SM, Fig. 4) hamstring avulsions^6,16,42-44^, with a highly predictable rate of return to pre-injury level of sports. In proximal non-retracted partial avulsions that remain symptomatic, MRI can show fluid between the ischial tuberosity and the injured tendon attachment, indicating an incomplete healing process. For these patients, surgery is equally also indicated, with a high rate of successful outcomes.^5,45^

**Fig. 5.**
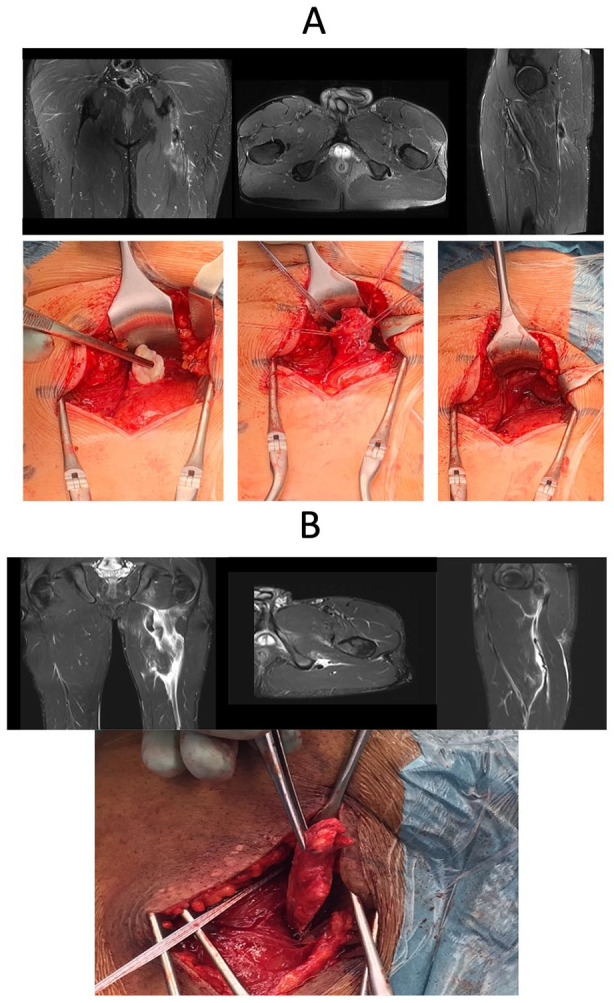
Isolated complete proximal single-tendon rupture: A) BF (MRI and perioperative images), B) SM (MRI and perioperative images).

In a proximal single-tendon avulsion with retraction from the ischial tuberosity, surgery is also recommended in high-level athletes regardless of which of the hamstring tendons is involved: BF (Fig. 5A), ST or SM (Fig. 5B).^6^ Without optimal treatment, permanent weakness and pain produce a suboptimal hamstring function.

#### Distal tendon

**Fig. 6.**
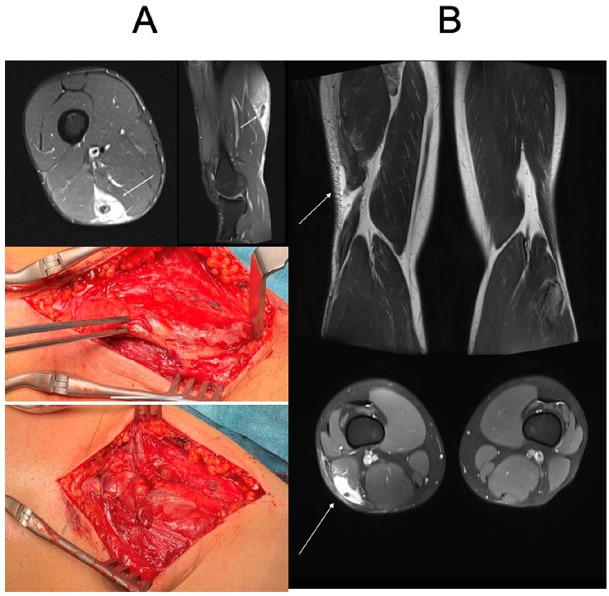
A) Distal tendon rupture of the ST (MRI and perioperative images before and after repair) and B) distal MTJ area rupture of the BF (MRI images, arrows indicate the retracted muscle belly and tendon stump).

Severe distal tendon injuries are rare compared with proximal hamstring tendon injuries. In athletes, in a complete distal tendon rupture surgery is usually indicated, and nonoperative management carries a high risk of failure.^21,46-48^ A complete distal rupture of BF or ST should be repaired anatomically as soon as possible after acute or acute-on-chronic injury^23,25,48,49^ (Figs. 2 and 6). Distal SM avulsion injuries are rare, with a marked negative impact on competitive sports participation. They are typically managed operatively.^23,50^

#### Central tendon

**Fig. 7.**
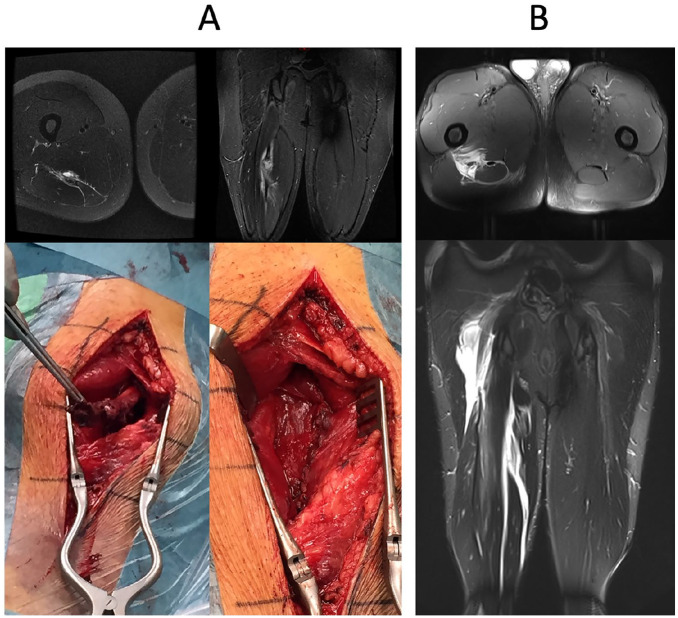
A) Central tendon rupture of the SM (recurrent injury, MRI and perioperative images before and after repair) and B) BF (acute injury, MRI).

Central or free (paramuscular) tendon injuries of the BF tend to become chronic and to recur despite appropriate conservative management^17^ (Fig. 7). In addition, recurrent central tendon injuries produce longer absence from play compared with other hamstring injuries. These central tendon injuries have a high risk of poor healing with nonoperative measures.^16^ If adequate nonoperative management following an acute injury has failed, and recurrent injuries ensue, surgery should be considered.

### Nonoperative treatment of proximal hamstring tendon avulsions

Complete proximal hamstring avulsions have a poor prognosis to heal without adequate treatment, especially in athletes. Many studies have been conducted to compare whether operative or nonoperative treatments are superior.^40,50-52^ However, it seems that these complete three-tendon avulsion injuries do not heal properly without anatomical repair, especially if there is a clear retraction from the ischial tuberosity.^39,53^ Only some case series have been published on individual proximal hamstring tendon injuries^54-56^ showing superior result of surgery in athletes. Nonoperative treatment of isolated proximal tendon ruptures in athletes has not been fully discussed in the literature. However, most of the hamstring injuries are muscle tissue strains or partial tears with good prognosis when treated by conservative means (modified rest, ice, and progressive rehabilitation).

### Discussion

The present article introduces the individual muscle-tendon concept in athletes. These injuries are prone to become disabling and chronic if misdiagnosed.^32^ Each hamstring muscle—BF, ST, and SM—has its own function and anatomy, and therefore should be considered individually. When BF, ST, and SM are considered separately and appropriate treatment administered, the risk of recurrent injury might be lower.

Hamstring injuries are typically related to soccer^9^, and recurrent injuries are common, often leading to substantial loss of play.^10^ Hamstring injuries can eventually jeopardize players’ careers. Based on our clinical observations, hamstring injuries should be managed according to which individual muscles and tendons are involved. Each hamstring muscle—BF, ST, and SM—has its own function, purpose, and injury pattern. If one of these three tendons is completely ruptured, it may permanently impair athlete’s performance and often also cause significant pain. These injuries should be probably managed surgically in top-level athletes. In practice, we should deal and manage each muscle and tendon of the hamstring complex individually, similar to what already happens in the gastrocnemius / soleus / Achilles muscle-tendon complex, and consider BF, ST, and SM and each of their tendons separately.

Different classifications of hamstring injuries are available.^4,57^ However, clinical and MRI data can contrast with each other.^58^ The MLG-R classification takes into consideration the mechanism of injury (M), location of injury (L), level of severity (G), and number of muscle re-injuries (R) and is based on an MRI. Although the return to play seems to somewhat correlate to the higher grade of injury, the choice of treatment should be considered individually.

Typically, avulsion of the three attachment tendons from the ischial tuberosity is considered an indication for surgery,^59^ but the treatment strategy with partial or single proximal tendon avulsion can be debated.^40,50^ In athletes, the loss of time to return to play and the rate of recurrence should be minimized. High-quality studies regarding individual hamstring tendon avulsion are scarce. Recently, Ayuob et al have published case series of operative treatment to avulsions of single tendon.^55^ These results are in line with other earlier studies.^6,54^ However, level 1 evidence is lacking as comparative studies do not exists in the current literature.

In conclusion, based on the present knowledge of anatomy and the different functions of each of the muscles of the posterior aspect of the thigh, and the capability of MRI to allow to formulate an accurate diagnosis, the term “hamstring injury” seems somewhat inaccurate. To improve the standard level of the treatment, especially in athletes, we should precisely identify which individual muscle(s) is(are) affected. Formulating a precise diagnosis would prompt to talk about BF, SM, or ST injury, or a combination of them. Complete single-tendon avulsions—BF, ST, SM, or their combined injury—in high-performance athletes could lead to a marked loss of function and chronic disability, and therefore operative treatment should often be considered.
